# Validity and reliability of the South African Triage Scale in prehospital providers

**DOI:** 10.1186/s12873-021-00406-6

**Published:** 2021-01-15

**Authors:** Nee-Kofi Mould-Millman, Julia M. Dixon, Taylor Burkholder, Jennifer L. Pigoga, Michael Lee, Shaheem de Vries, Kubendhren Moodley, Maxene Meier, Kathryn Colborn, Chandni Patel, Lee A. Wallis

**Affiliations:** 1 Department of Emergency Medicine, University of Colorado Denver, School of Medicine, Anschutz Medical Campus, 12631 E 17th Ave, Room 2612, MS C326, Aurora, CO 80045 USA; 2 Division of Emergency Medicine, University of Cape Town, Department of Surgery, Cape Town, South Africa; 3 University of Southern California, Keck School of Medicine, Los Angeles, California USA; 4 Western Cape Government, Department of Health, Emergency Medical Services, Cape Town, South Africa; 5 Department of Pediatrics, University of Colorado Denver, School of Medicine, Aurora, CO USA; 6 Department of Surgery, University of Colorado Denver, School of Medicine, Aurora, CO USA

**Keywords:** EMS, SATS, South Africa triage scale, Triage, Prehospital

## Abstract

**Background:**

The South African Triage Scale (SATS) is a validated in-hospital triage tool that has been innovatively adopted for use in the prehospital setting by Western Cape Government (WCG) Emergency Medical Services (EMS) in South Africa. The performance of SATS by EMS providers has not been formally assessed. The study sought to assess the validity and reliability of SATS when used by WCG EMS prehospital providers for single-patient triage.

**Methods:**

This is a prospective, assessment-based validation study among WCG EMS providers from March to September 2017 in Cape Town, South Africa. Participants completed an assessment containing 50 clinical vignettes by calculating the three components — triage early warning score (TEWS), discriminators (pre-defined clinical conditions), and a final SATS triage color. Responses were scored against gold standard answers. Validity was assessed by calculating over- and under-triage rates compared to gold standard. Inter-rater reliability was assessed by calculating agreement among EMS providers’ responses.

**Results:**

A total of 102 EMS providers completed the assessment. The final SATS triage color was accurately determined in 56.5%, under-triaged in 29.5%, and over-triaged in 13.1% of vignette responses. TEWS was calculated correctly in 42.6% of vignettes, under-calculated in 45.0% and over-calculated in 10.9%. Discriminators were correctly identified in only 58.8% of vignettes. There was substantial inter-rater and gold standard agreement for both the TEWS component and final SATS color, but there was lower inter-rater agreement for clinical discriminators.

**Conclusion:**

This is the first assessment of SATS as used by EMS providers for prehospital triage. We found that SATS generally under-performed as a triage tool, mainly due to the clinical discriminators. We found good inter-rater reliability, but poor validity. The under-triage rate of 30% was higher than previous reports from the in-hospital setting. The over-triage rate of 13% was acceptable. Further clinically-based and qualitative studies are needed.

**Trial registration:**

Not applicable.

**Supplementary Information:**

The online version contains supplementary material available at 10.1186/s12873-021-00406-6.

## Background

Medical triage is the process of systematically sorting patients based on acuity and anticipated resource need [1, 2]. Triage facilitates delivery of timely, quality care by mobilizing the right type of care for the right patient at the right time [3]. In a patient experiencing an acute stroke or myocardial infarction, for example, triage performed by emergency medical services (EMS) providers may allow earlier prehospital recognition of the acute condition thereby triggering faster delivery of appropriate pre- and in-hospital care to help minimize morbidity and mortality [1–3].

Several in-hospital triage tools exist that are commonly used to triage undifferentiated patients on arrival to emergency departments, many with demonstrated clinical and operational benefits. In the prehospital setting, a singular, internationally-accepted tool or system for the initial triage of undifferentiated emergent patients in the field by EMS providers does not exist [1–3].

A 2018 systematic review of in-hospital adult emergency care triage tools used in low-and-middle income countries concluded that the South African Triage Scale (SATS) had the highest quality of evidence with sensitivity and specificity of 70–75% and 91–97%, respectively [4]. Additionally, a prior South African emergency center study found SATS had an over-triage rate of 15% and under-triage rate of 10% [2]. SATS was originally developed in 2006, created for and validated amongst in-hospital emergency care physicians and nurses in South Africa [5–8]. To use SATS, a numerically-based Triage Early Warning Score (TEWS) is first calculated from the total of a numerical score to each of five vital signs, mobility and trauma and can range from 0 to 17. A Score of 0, 1, or 2 is assigned green; 3 or 4 is yellow, 5 or 6 is orange and 7 or greater is red. If a “discriminator” (i.e., a high-risk clinical condition such as chest pain or current seizure) is present the patient’s triage color is upgraded to match the category assigned to each clinical discriminator in the SATS reference table (see Additional file 1). The final SATS colors used to denote triage acuity and priority, from highest to lowest acuity, are: Red, Orange, Yellow, and Green; Blue is dead) [2, 6, 9, 10].

In 2012, SATS was incorporated into routine prehospital emergency care use by the Western Cape Government (WCG) EMS system. The WCG EMS system is a public EMS system that provides 24/7 ambulance services to a catchment population of over 6 million in the Western Cape Province of South Africa [11]. In 2017, WCG EMS employed approximately 2000 operational EMS providers in across three cadres: basic, intermediate, and advance life support (BLS, ILS, and ALS, respectively); they executed approximately 450,000 ambulance responses, and providers are expected to use SATS in all clinical cases [11, 12].

Although all cadres of EMS providers have the skills and tools to derive the SATS triage score in an ambulance, SATS was not intended for, nor formally adapted to, prehospital emergency care [9]. To date, the prehospital triage performance characteristics of SATS remain unstudied. Accurate prehospital triage is necessary to minimize under-triage which can lead to inadequate intervention or transport to lesser-equipped facilities, and over-triage which can result in wasteful and harmful unnecessary interventions or transport to over-burdened tertiary facilities [13].

## Methods

### Aim

The objective of this study is to assess the inter-rater reliability (i.e., consistency among raters) and validity (i.e., triage accuracy) of the SATS when used by WCG EMS prehospital providers for single-patient triage.

### Design

The study was designed as a prospective, assessment-based validation study among WCG EMS providers from March to September, 2017 in Cape Town, South Africa.

### Setting and participants

At the time of this study, foundational education for WCG EMS providers from across the Western Cape Province included a 6-week certificate courses for BLS (recently discontinued), a 12-week course for ILS (soon to be replaced with a 1-year certificate), and a 2-year (diploma) and 4-year (degree-earning) training for ALS providers [14]. WCG EMS providers responded to over 500,000 calls per year, of which approximately 40% are trauma cases [11, 12]. Providers often staff ambulances as a mixed-tier crew (e.g., BLS with ILS, or BLS with ALS). BLS providers’ scope of practice is limited and best described as advanced first aid (e.g., airway suctioning, splints and wound care) plus cardiopulmonary resuscitation with general access to a relatively narrow selection of medications (e.g., oxygen, oral glucose and oral non-opioid analgesics). ALS providers, however, can deliver a wide variety of drugs and may perform advanced cardiac, trauma, and critical care life support and procedures, including endotracheal intubation and ventilator management. ILS providers’ scope of practice lies between BLS and ALS, and ILS providers can perform several invasive interventions and deliver a narrow selection of intravenous drugs [12].

Initial training on the use of SATS occurred “on the job” by educators within the EMS system – however, all cadres of providers received the same SATS training. WCG EMS providers intermittently may participate in various refresher trainings, short courses, and advancement courses which are offered at the Western Cape Province College of Emergency Care several times per year. Topics at the College cover a variety of clinical and non-clinical content (e.g., leadership training). All providers enrolled in courses at the College during the study period were eligible to participate in this study. One BLS, one ILS, and one ALS cohort each at the College was chosen from which study participants would be enrolled.

### Recruitment

From March to September 2017, study investigators recruited a convenience sample of 102 participants from the College. Sample size calculations were based on an intraclass correlation coefficient (ICC; a descriptive statistic used when quantitative measurements are made on units that are organized into groups) of 0.2, power of 0.9, and alpha set of 0.05. None of the participants were actively enrolled in courses at the College that pertained to triage nor use of SATS [15]. Participants from classroom cohorts of BLS, ILS and ALS providers were approached for consent. Staff at the College of Emergency Care delivered advanced advertisement of the study to eligible classes. A study staff member verbally reviewed informed consent with all potential participants, and written consent was obtained from each willing participant.

### Assessment

**Fig. 1:**
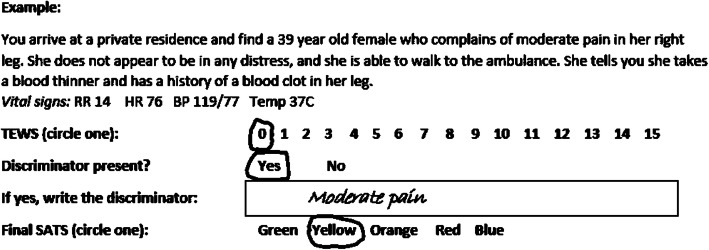
Sample vignette from the SATS written assessment

Participants were briefed on the assessment procedures by a study investigator (JP, TB). Participants were individually administered the written SATS assessment under supervision of one study investigator. Participants were each given an examination booklet and a standard SATS adult color reference table (routinely available in their clinical practice) (online Additional file 1). Each assessment was administered in an examination booklet, comprised of 50 clinical vignettes of adult prehospital emergency cases typical for the Western Cape Province (see sample vignette in Fig. 1). The 50 vignettes were retrieved from a larger set of 100 validated vignettes previously used for hospital staff assessment of SATS [2]. Vignettes were purposefully selected to represent a mixture and balance of case types and SATS distributions that are representative of those seen by WCG EMS. The case context in the original hospital-based vignettes were lightly edited by study investigators (NM, TB, JD) to reflect the prehospital context, but the core clinical scenario and gold standard TEWS, discriminator, and final SATS were unchanged [10–14]. Twomey and co-investigators from the hospital-based study re-scored all 50 vignettes by re-providing gold standard TEWS, discriminators, and SATS answers for each vignette. There were 19 trauma and 31 medical vignettes. Selected vignettes had the following gold standard SATS color (i.e., final triage color): 5 green, 15 yellow, 23 orange, 6 red and 1 blue. By SATS convention, blue is dead on arrival, red denotes the highest triage acuity, followed by orange, yellow and green corresponds to the lowest acute). The correct TEWS responses in vignettes ranged from 0 to 13. See Additional file 2 for all vignettes.

### Data collection

Each participant provided demographic information (age, sex, qualification, years of experience, and current district). For each clinical vignette, participants provided a TEWS value (between 0 and 17), a clinical discriminator (if applicable), and the final SATS color. Responses were manually entered into a password-protected Microsoft Excel spreadsheet Version 15.0 (Microsoft Corporation, Redmond, Washington, USA) by a study staff member. A second research team member manually verified accurate data entry from a random selection of 10% of assessments.

### Analysis

Reliability was assessed by inter-rater reliability, defined as level of agreement in vignette item (i.e., TEWS, discriminators, and SATS) responses among EMS providers. Validity (i.e., triage accuracy) was assessed using mistriage rates, defined as rates of over- or under-triage when comparing EMS raters to gold standard vignette responses.

Cleaned data were exported from the Microsoft Excel spreadsheet into a statistical software program R, version 3.4.0 (R Core Team, Vienna, Austria). Demographic data were descriptively analyzed. Vignette responses were scored in comparison to the gold standard answers to calculate the proportions of TEWS or SATS that were under, over or accurately determined (e.g., a score of Orange is considered under triage if the gold standard score is Red), and the proportion of discriminators that were missing, correct or incorrect.

A novel statistical measure of agreement, Sklar’s Omega, was used. Sklar’s Omega is a Gaussian copula-based framework that permits estimation of the degree of agreement between the EMS providers’ scores and the gold standard physicians’ scores [16]. Confidence intervals were estimated using bootstrapping methods with 1000 iterations. Traditional interpretations of agreement were used: less than 0.2 represented slight agreement, between 0.2 and 0.4 fair agreement, between 0.4 and 0.6 moderate agreement, between 0.6 and 0.8 substantial agreement, 0.8 or greater was considered near-perfect agreement [17]. The absolute difference between the overall expected agreement with gold standard and inter-rater was averaged to find an overall average difference for each vignette.

## Results

The assessment was completed by 102 WCG EMS providers with mean age of 35-years (SD 7.7) and mean field experience of 6.3-years (SD 5.5) (Table 1). A total of 5100 vignette responses (i.e., 50 vignettes × 102 EMS provider respondents) were available for analysis.

**Table 1:** Western Cape Government EMS provider characteristics (*N* = 102)

Characteristics	Participants, n (%)
Sex	
Male	50 (49)
Female	41 (40)
Missing	11 (11)
Qualification	
BLS	59 (58)
ILS	37 (36)
ALS	6 (6)
Location of Practice	
Rural	42 (41)
Metropole	52 (51)
Missing	8 (8)
Mean age (SD; range)	35 years (7.8; 21–55)
Mean Field Experience (SD; range)	6.3 years (5.5; 0.9–30.2)

*ALS* advanced life support, *BLS* basic life support, *ILS* intermediate life support, *SD* standard deviation

Overall, the final SATS triage color was accurately determined in 2883 (56.5%) vignette responses. The under-triage rate was 29.5%, and the over-triage rate was 13.1% (Table 2). The highest proportion of under-triage occurred in vignettes with gold standard SATS of red (300, 49%), and over-triage most often occurred in vignettes with a gold-standard SATS of green (213, 41.8%). There were similar proportions of under-triage in trauma (559, 28.8%) and medical vignettes (947, 30.0%) (Table 2). The over-triage rate was also similar in medical (400, 12.7%) and trauma (266, 13.7%) cases.

**Table 2:** Comparison of participant and gold standard vignette TEWS, discriminator, and SATS responses

**SATS Color vignette responses**^**a**^					
	**Correct** **(%)**	**Under-Triaged (%)**	**Over-Triaged (%)**	**Missing** **(%)**	**Total** **(%)**
**Overall**	2883 (56.5)	1506 (29.5)	666 (13.1)	45 (0.9)	5100 (100)
**Gold Standard**					
**Red**	307 (50.1)	300 (49.0)	n/a	5 (0.8)	612 (100)
**Orange**	1268 (54.0)	867 (37.0)	189 (8.1)	22 (0.9)	2346 (100)
**Yellow**	918 (60.0)	334 (21.8)	264 (17.2)	14 (0.9)	1530 (100)
**Green**^**b**^	294 (57.7)	n/a	213 (41.8)	3 (0.6)	510 (100)
**Blue**^**c**^	96 (94.1)	5 (4.9)	n/a	1 (1.0)	102 (100)
**Trauma**	1098 (56.7)	559 (28.8)	266 (13.7)	15 (0.8)	1938 (100)
**Medical**	1785 (56.4)	947 (30.0)	400 (12.6)	30 (1.0)	3162 (100)
**TEWS vignette responses**^**a**^					
	**Correct** **(%)**	**Under-Calculated (%)**	**Over-Calculated (%)**	**Missing** **(%)**	**Total** **(%)**
**Overall**	2173 (42.6)	2296 (45.0)	555 (10.9)	76 (1.5)	5100 (100)
**Gold Standard**					
**Red**	107 (17.5)	482 (78.8)	21 (3.4)	3 (0.3)	612 (100)
**Orange**	1103 (47.0)	915 (39.0)	299 (12.8)	29 (1.2)	2346 (100)
**Yellow**	653 (42.7)	670 (43.8)	189 (12.4)	18 (1.2)	1530 (100)
**Green**	306 (60.0)	158 (31.0)	41 (8.0)	5 (1.0)	510 (100)
**Blue**	4 (3.9)	48 (47.1) ^d^	28 (27.5)	22 (21.6)	102 (100)
**Trauma**	569 (39.4)	1141 (58.9)	184 (9.5)	44 (2.3)	1938 (100)
**Medical**	1604 (50.7)	1155 (36.5)	371 (11.7)	32 (1.0)	3162 (100)

^a^102 respondents each completing a 50-vignette assessment = 5100 vignette responses in total

^b^Green patients are lowest acuity, so under-triage is not possible

^c^Blue patients are dead, so over-triage is not possible

^d^Includes 43 vignette responses in which TEWS was scored as 0

Compared to the gold standard, the TEWS score was correctly calculated in 2173 (42.6%), under-calculated in 2296 (45.0%), and over-calculated in 555 (10.9%) of all vignette responses (*n* = 5100) (Table 2). TEWS was most often incorrect in trauma cases (1325, 68.4%), mostly attributable to under calculating the score in 1141 (58.9%) of trauma vignette responses.

TEWS was most often under-calculated in vignettes with a final SATS of red (482, 78.8%) and over-calculated in cases with gold standard SATS of orange (299, 12.8%), yellow (189, 12.4%), and green (41, 8.0%), but least-often over-calculated in cases with gold standard SATS of red (21, 3.4%).

**Fig. 2:**
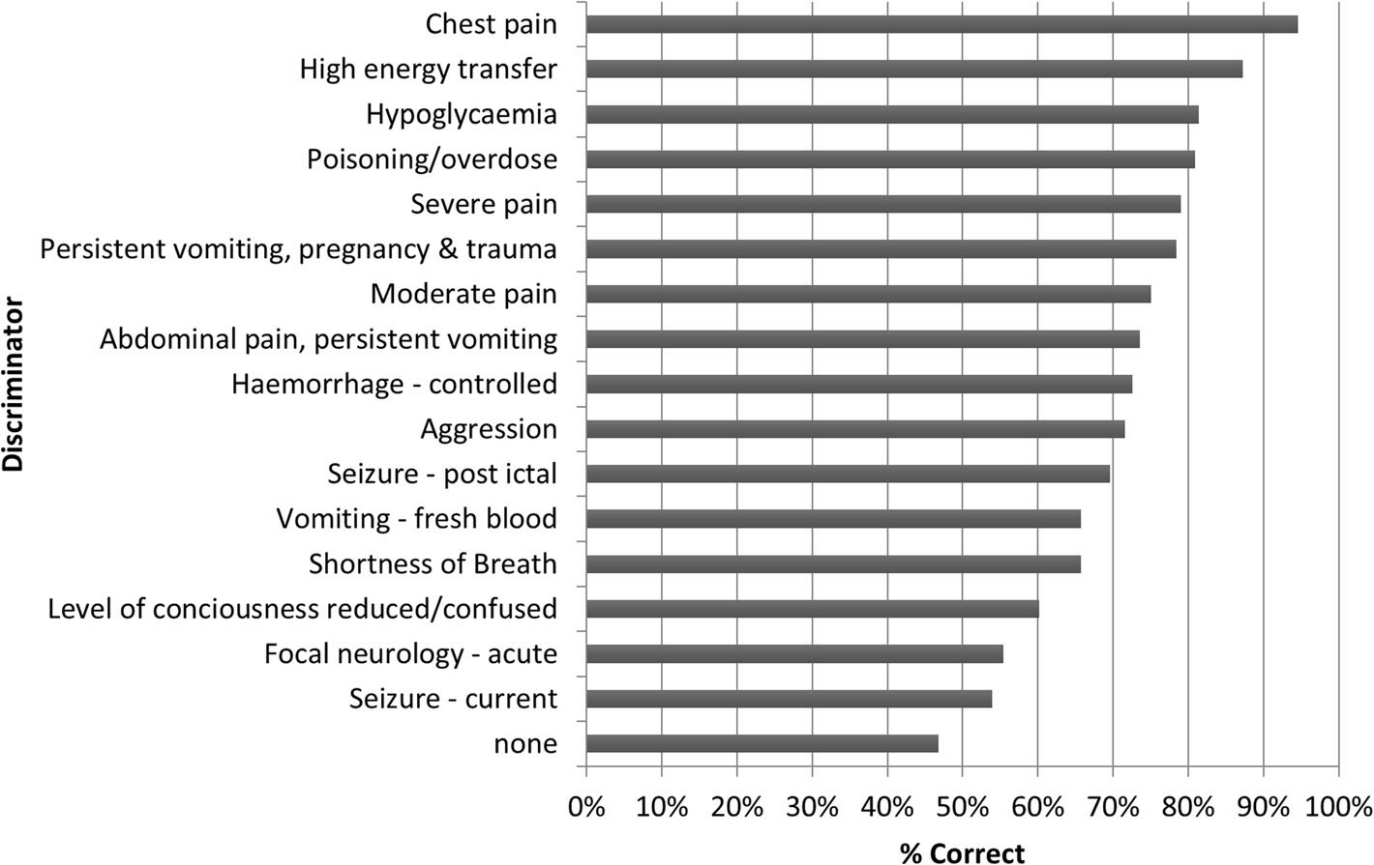
Accuracy of discriminators used in vignettes

Out of 50 vignettes, 42 (84%) had at least one discriminator which was correctly identified (yes/no) in 3570 (70.0%) of all 5100 vignette responses (Table 3). Further, the specific discriminator was correctly listed in 2521 (58.8%) of applicable vignette responses (Table 3). Of the 1506 vignette responses that were under-triaged, the selected clinical discriminator was seldom correct (392, 26.0%) and very often incorrect (1114, 74.0%). The discriminator was incorrect or missing in 1017 (52.5%) of trauma and in 1526 (48.3%) of medical vignette responses. The clinical discriminator was correctly identified for 183 (29.9%) of red, 1231 (52.5%) of orange, 826 (54.0%) of yellow and 242 (47.5%) of green SATS gold-standard cases. The most frequent error regarding use of discriminators occurred in cases where no discriminator was expected (per gold standard) of which participants selected that a discriminator was indicated in 360 (44.1%). High energy transfer, burn circumferential and reduced level of consciousness had the lowest percentages of correct discriminator use, with 31 (15.2%), 18 (17.6%) and 54 (17.6) of relevant vignette responses, respectively (Fig. 2).

**Table 3:** Use of discriminator in vignettes

Respondents’ answer correctly indicated that:	Vignettes in which final SATS was:				All Cases (%)
	Correct (%) ***N*** = 2883	Under-Triaged (%) ***N*** = 1506	Over-Triaged (%) ***N*** = 666	Missing (%)^**a**^	
**A discriminator was, or was not, needed**	2309 (80.1)	851 (56.9)	379 (56.9)	31 (0.9)	3570 (70.0)^b^
**Discriminator was correctly explained**	1912 (66.3)	392 (26.0)	202 (30.3)	15 (0.6)^c^	2521 (58.8)^c^

Row percentages do not add to 100% because multiple vignettes had more than 1 discriminator

^a^Missing % = n from missing / n from row total

^b^*N* = 5100 total vignette responses (i.e., 50 vignettes each with 102 respondents)

^c^*N* = 4284 total eligible vignette responses (i.e., 42 vignettes each with 102 respondents)

Table 4 summarizes the results of the agreement calculations. Inter-rater agreement among prehospital providers was consistently stronger than agreement between providers and gold standard when assessing SATS, TEWS, and the discriminator. Overall, there was substantial inter-rater and gold standard agreement for TEWS that ranged from moderate to substantial. SATS performed similarly with substantial agreement for both inter-rater and providers’ agreement with gold standard. Discriminator agreement was moderate for inter-rater and gold standard comparisons.

**Table 4:** Agreement among providers and agreement between providers and gold standard

	Inter-Rater Agreement Mean (95% CI)	Agreement with Gold Standard Mean (95% CI)
**TEWS**	0.690 (0.552, 0.781)	0.677 (0.557, 0.783)
**SATS**	0.710 (0.577, 0.804)	0.695 (0.588, 0.791)
**Discriminator**	0.589 (0.440, 0.712)	0.491 (0.371, 0.592)

*TEWS* Triage Early Warning Score, *SATS* South African Triage Scale

Table 5 summarizes the top 5 most influential vignettes on the total inter-rater agreement and agreement between providers and gold standard with regard to the final triage score (i.e., SATS color). The left-most column is the number of the vignette being left out during that calculation of agreement. The middle column describes the case in the vignette. The right-most column is the average difference in Sklar’s Omega when that vignette was left out of the estimation. From this table, vignette #17 has the most influence on final SATS agreement, followed by vignettes 37, 47, 6, and 20. Four are medical cases, and one is a trauma case. All are low acuity cases (i.e., gold standard SATS of green).

**Table 5:** Influential Vignettes

Vignette	Brief description of Vignette	Averaged Difference^**a**^
**17**	Medical case – low acuity, psychiatric illness with agitation	0.049
**37**	Trauma case – low acuity, ground level fall with arm pain	0.048
**47**	Medical case – low acuity, severe pain from buttock abscess	0.048
**6**	Medical case – low acuity, severe pain from abdominal complaint	0.047
**20**	Medical case – low acuity, asthma exacerbation	0.047

^a^Average difference in Sklar’s Omega when the vignette was left out of the estimation

## Discussion

SATS is a well-established in-hospital emergency care triage tool used in South Africa and multiple other low- and middle-income countries. This study is the first formal assessment of SATS among a cohort of EMS providers with prehospital experience using SATS. We found that SATS had poor validity, evidenced by high rates of under-triage, and moderate inter-rater reliability, evidenced by consistent mis-triage among EMS providers.

Overall, SATS underperformed as a prehospital triage tool - the final SATS triage color was only correct in about one-half (57%) of cases and there was a high (30%) rate of under-triage. However, the over-triage rate was 13% which we considered acceptable, but not excellent. We based this conclusion from a report by Twomey et al. who reported that an under-triage rate of 10% and an over-triage rate of 15% were adequate when SATS was tested among South Africa in-hospital emergency physicians and nurses [2]. In their report from 2012, Twomey et al. compared those rates to the only existing and accepted international pre- and in-hospital triage rates from the American College of Surgeons Committee on Trauma which recommends an under-triage rate of 5–10% and an over-triage rate below 50% for prehospital trauma triage in the USA [18]. Considering that SATS triages conditions beyond trauma, and considering the resource-limitations of the South African health system, Twomey et al. concluded that 15 and 10% SATS over- and under-triage rates, respectively, were acceptable [2]. The very high under-triage rate in this prehospital study appears to be due to under-calculation of TEWS and incorrect use of clinical discriminators by EMS providers. The medical implication is that the acute patients may be often under-triaged, which may be medically harmful as patients may be transported to hospitals with lower levels of care, may not receive the requisite level of ambulance care or may experience delays in care.

TEWS was often under-calculated. TEWS is an accurate predictor of need for emergency treatment, prognosis of emergency patients and identifying patients at risk for adverse outcomes. Calculation of TEWS requires two steps: first, assigning points to five physiologic parameters (respiratory rate, heart rate, systolic blood pressure, temperature, and mental status), mobility status, and trauma status; and second, adding all points to yield the total TEWS [19]. TEWS was very often under-calculated in trauma cases and high-acuity (“red”) cases. Higher acuity patients, by definition, have more deranged vital signs which theoretically increases the complexity of calculating a TEWS, thereby increasing the likelihood of a computational error in the two-step process. Since these paper-based vignettes were administered in a relaxed classroom setting with a reference sheet, the component of provider stress that could further exacerbate computational errors in real life clinical care was likely significantly minimized, implying that under-computations of TEWS could likely be worse in real life application during critical cases. Explanations for why TEWS was often incorrect in trauma cases may be due to errors of commission (e.g., providers’ incorrectly assigned points for injury and/or the mobility statuses) or omission (e.g., providers forgot that trauma and/or mobility statuses require the addition of points) – the specific reason requires further investigation.

The clinical discriminators were frequently incorrectly selected, or very often missing when one was indicated, which occurred uniformly across medical and trauma case types, and more frequently incorrect in red cases. This contributed to the overall high under-triage rate. The clinical discriminators were originally developed by the Cape Triage Group in 2006 to help identify high-risk emergency conditions that present to in-hospital front-line clinicians (emergency doctors and nurses) in South Africa [5, 19]. Many of these clinical conditions may be difficult for EMS providers to consistently apply as many discriminators are subjective (e.g., ‘moderate pain’ or ‘high energy transfer’), require diagnostic information (e.g., ‘hypoglycemia’ or ‘dislocation’) or may be challenging to accurately establish in the field (e.g., ‘burn over 20%’ or ‘poisoning/overdose’). Since the clinical discriminators can over-ride the triage color determined by the TEWS score, the discriminator alone can dictate the final SATS color independent of TEWS. The study found that several trauma-relevant discriminators e.g., ‘high energy transfer’, ‘burn circumferential’ and ‘haemorrhage controlled’, and neurologic discriminators (e.g., ‘level of consciousness reduced/confused’ and ‘focal neurologic’ issue) were among the most frequently incorrectly applied discriminators. Improving accurate use of trauma- and neurologic-relevant discriminators may require supplemental EMS provider training and/or modification of those discriminators so they are more EMS provider appropriate. However, overall, the clinical discriminators – as originally designed – did not perform well in this cohort of EMS providers.

It was interesting to find that SATS was used reliably. Specifically, we found substantial inter-rater agreement in determination of the final SATS triage color (k = 0.71), which indicates consistency in application among EMS providers. A prior reliability study of South African in-hospital providers found comparable overall SATS kappa scores indicating substantial agreement among nurses (k = 0.66) and among physicians (k = 0.76) [9]. Of note, use of clinical discriminators had relatively worse agreement, both within EMS providers (k = 0.59) and when EMS providers were compared to gold standard (k = 0.49). These findings suggest that EMS providers, as a cohort, use this triage tool fairly inconsistently compared to in-hospital providers, but use it consistently within themselves – however, we should note that the EMS providers generated incorrect triage scores 43% of the time, as a cohort, due to incorrect calculation of TEWS and/or misapplication of the clinical discriminators. This suggests there are consistent errors (resulting in poor accuracy) in how these EMS providers use SATS, often attributable to the discriminators.

### Recommendations

Based on our findings, there are several key recommendations. First, there exists an opportunity for focused re-training of prehospital providers on clinical discriminators, with emphasis on trauma and neurologic complaints and several other frequently misused discriminators. Second, EMS providers may also benefit from re-training and reminders to help them consider the patient’s trauma and ambulatory status during calculation of the TEWS; however, this may be qualification dependent. Third, EMS providers may need computational assistance with calculation of the TEWS, and/or a re-formulation of the TEWS table to minimize the likelihood of a computational error, especially in critical (‘red’) cases. Last, the clinical discriminators may need re-formulation to be more compatible with prehospital providers’ clinical knowledge, ‘diagnostic’ capabilities and clinical context.

### Limitations

This study utilized a written assessment rather than simulated cases or chart analysis and therefore may not reflect the true performance of SATS in the ‘live’ clinical environment. However, the artificiality of the testing environment should not affect inter-rater reliability, as the effect of the testing setting will be distributed between the group of subjects and within individual subjects. Additionally, the vignettes tested some, but not all, of the clinical discriminators or combinations of TEWS values, so findings are limited to only those tested. Analysis of results according to level of training ALS, ILS or BLS was not done due to the relatively small sample size.

## Conclusions

This study was the first assessment of validity and inter-rater reliability of prehospital SATS triage among a cohort of South African EMS providers. Overall, SATS under-performed as a prehospital triage tool. The study found good reliability, but poor validity, among EMS providers using SATS for prehospital triage in clinical vignettes. The final SATS triage color was correctly determined in only about one-half of cases. The under-triage rate of 30% was higher than previous reports from the in-hospital setting. The over-triage rate of 13% was acceptable. The high under-triage rate of SATS is attributable to under-calculation of TEWS and incorrect use of clinical discriminators. Discriminators could be better tailored to prehospital medicine. Additional clinical and qualitative studies of EMS providers are needed to fully understand the performance and use of SATS.

## Supplementary Information

**Additional file 1.**

**Additional file 2.**

## Data Availability

The datasets used and/or analysed during the current study are not publicly available but are available from the corresponding author on reasonable request.

## Abbreviations

ALS: Advanced Life Support
BLS: Basic Life Support
EMS: Emergency Medical Services
ILS: Intermediate Life Support
SATS: South Africa Triage Scale
SD: Standard Deviation
TEWS: Triage Early Warning Score
WCG: Western Cape Government

## References

[1] Mistry B, Stewart De Ramirez S, Kelen G (2018). Accuracy and reliability of emergency department triage using the emergency severity index: an International Multicenter Assessment. Ann Emerg Med.

[2] Twomey M, Wallis LA, Thompson ML, Myers JE (2012). The south African triage scale (adult version) provides reliable acuity ratings when used by doctors and enrolled nursing assistants. Afr J Emerg Med.

[3] Fernandes CM, McLeod S, Krause J (2013). Reliability of the Canadian triage and acuity scale: interrater and intrarater agreement from a community and an academic emergency department. CJEM.

[4] Jenson A, Hansoti B, Rothman R, de Ramirez SS, Lobner K, Wallis L (2018). Reliability and validity of emergency department triage tools in low- and middle-income countries: a systematic review. Eur J Emerg Med.

[5] Gottschalk SB, Wood D, DeVries S, Wallis LA, Bruijns S (2006). The cape triage score: a new triage system South Africa. Proposal from the cape triage group. Emerg Med J.

[6] Rosedale K, Smith ZA, Davies H, Wood D (2011). The effectiveness of the south African triage score (SATS) in a rural emergency department. S Afr Med J.

[7] Sunyoto T, Van den Bergh R, Valles P (2014). Providing emergency care and assessing a patient triage system in a referral hospital in Somaliland: a cross-sectional study. BMC Health Serv Res.

[8] Dalwai MK, Twomey M, Maikere J (2014). Reliability and accuracy of the south African triage scale when used by nurses in the emergency department of Timergara Hospital, Pakistan. S Afr Med J.

[9] Twomey M, Wallis LA, Thompson ML, Myers JE (2012). The south African triage scale (adult version) provides reliable acuity ratings. Int Emerg Nurs.

[10] Cheema B, Twomey M. In: Health Do, editor. The South Africa Triage Scale (SATS), Training Manual 2012: Emergency Medicine Society of South Africa. Cape Town: Western Cape Government Department of Health; 2012. https://emssa.org.za/wp-content/uploads/2011/04/SATS-Manual-A5-LR-spreads.pdf.

[11] Western Cape Government. Medical Emergency Transport and Rescue. Western Cape Government. https://www.westerncape.gov.za/service/medical-emergency-transport-and-rescue-metro#:~:text=If%20you're%20are%20dialling,Cape%20Town%20General%20Emergency%3A%20107. Published 2019. Updated 22 November 2019. Accessed 09/04/2020, 2020.

[12] Mould-Millman NK, Dixon J, Lamp A (2019). A single-site pilot implementation of a novel trauma training program for prehospital providers in a resource-limited setting. Pilot Feasibility Stud.

[13] Sasser SM, Hunt RC, Faul M (2012). Guidelines for field triage of injured patients: recommendations of the National Expert Panel on Field Triage, 2011. MMWR Recomm Rep.

[14] Sobuwa S, Christopher L (2019). Emergency care education in South Africa: past, present and future. Aust J Paramed.

[15] Kings M (2016). Personal Communication with Michele Kings from the Western Cape College of Emergency Care.

[16] Hughes J. SKLAR’s omega: a gaussian copula-based framework for assessing agreement. Cornell University; 2018. arXiv preprint arXiv:180302734.

[17] Munoz SR, Bangdiwala SI (1997). Interpretation of kappa and B statistics measures of agreement. J Appl Stat.

[18] American College of Surgeons Committee on Trauma (2006). Resources for optimal care of the injured patient 2006.

[19] South Africa Triage Group. Adult SATS chart: Emergency Medicine Society of South Africa. https://emssa.org.za/wp-content/uploads/2011/04/SATS-Manual-A5-LR-spreads.pdf. Published 2012. Accessed 9/11/2020; 2020.

