# Supplementary figures and images for: Validity and reliability of the South African Triage Scale in prehospital providers
Source: BMC Emerg Med. 2021 Jan 15;21:8. doi: 10.1186/s12873-021-00406-6 (PMC7811258; doi:10.1186/s12873-021-00406-6)

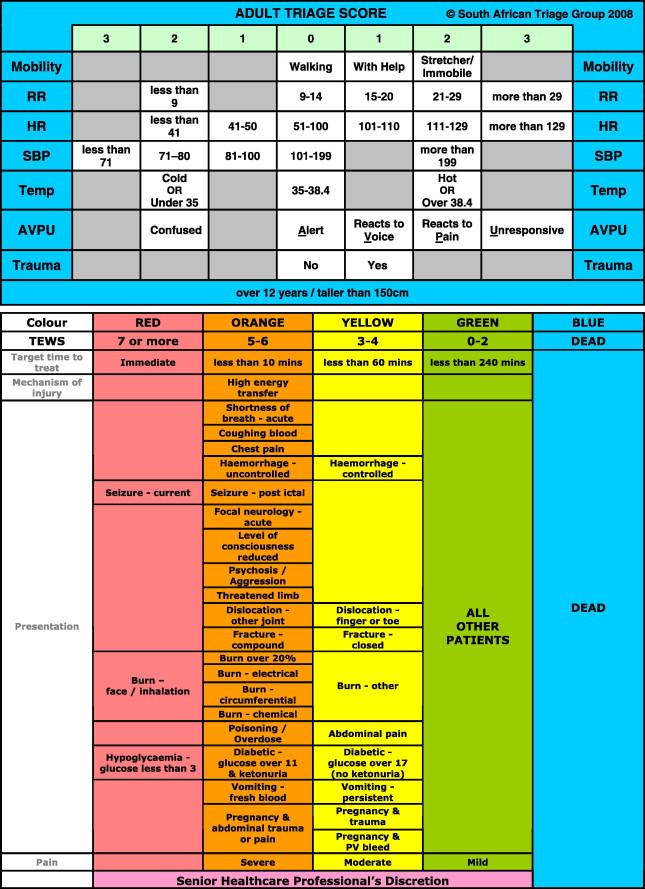

Supplement: Supplementary file 1 — Additional file 1. [file 12873_2021_406_MOESM1_ESM.jpg]
