# Supplementary material for: Validity and reliability of the South African Triage Scale in prehospital providers
Source: BMC Emerg Med. 2021 Jan 15;21:8. doi: 10.1186/s12873-021-00406-6 (PMC7811258; doi:10.1186/s12873-021-00406-6)
Supplement: Supplementary file 2 — Additional file 2. [file 12873_2021_406_MOESM2_ESM.pdf]

Assessing use of the South African Triage Scale (SATS) by Western Cape Government EMS

THANK YOU FOR PARTICIPATING! PLEASE READ:

- We are studying the use of the South African Triage Scale (SATS) in the Western Cape prehospital setting.
- We are asking for your help because you have some valuable knowledge and experience on this topic.
- In this written assessment, you will assign a TEWS score, discriminator, and SATS colour to each case.
- We will perform an anonymous analysis on all the written answers to see how the SATS performs.
- Your participation is voluntary and you have the right to refuse to be in this study. You can stop at any time after giving your consent. This decision will not negatively affect your employment in any way.

Your answers and score on this assessment are **strictly confidential** and for research purposes only.

PLEASE COMPLETE THE FOLLOWING:

Your age: __________years

Gender (circle one): Male Female

Current qualification (circle one): BAA AEA CCA ECT BTech/BEMC _____

Duration of field experience: ____ years and _____ months

Your current district (circle one):

West Coast	Cape Winelands	Eden
Central Karoo	Overberg	Metropole South
Metropole East	Metropole North	Metrpole West

FOR EACH OF THE FOLLOWING PATIENT VIGNETTES PLEASE:

- Calculate and circle the correct TEWS
- Indicate if you would use a discriminator in this case (yes or no)
- If yes, which discriminator would you use? Write it
- Calculate the correct (final) SATS colour

YOU MAY USE THE ADULT SATS TABLE PROVIDED TO HELP YOU COMPLETE YOUR RESPONSES.

EXAMPLE:

You arrive at a private residence and find a 39 year old female who complains of moderate pain in her right leg. She does not appear to be in any distress, and she is able to walk to the ambulance. She tells you she takes a blood thinner and has a history of a blood clot in her leg.

Vital signs: RR 14 HR 76 BP 119/77 Temp 37C

TEWS (circle one): 0 1 2 3 4 5 6 7 8 9 10 11 12 13 14 15

Discriminator present? Yes No

If yes, write the discriminator: Moderate pain

Final SATS (circle one): Green Yellow Orange Red Blue

BEGIN VIGNETTES:

1. You are called to the scene of a 59 year old male with a known history of epilepsy. According to bystanders the patient just had a seizure. Currently, he only responds to pain.
Vital signs: RR 25 HR 163 BP 150/81 Temp 36.4°C HGT 2.8

TEWS (circle one): 0 1 2 3 4 5 6 7 8 **9** 10 11 12 13 14 15

Discriminator present? **Yes** No

If yes, write the discriminator: Hypoglycaemia

Final SATS (circle one): Green Yellow Orange **Red** Blue

---

2. You are called to an attempted suicide and respond to a patient laying on the ground outside a building where an adult man jumped out of a fourth floor window. There is blood on his face and around both legs. He complains of severe pain in his whole body and appears to have multiple fractures of his lower limbs. You immobilize him on spinal board.
Vital signs: RR 20 HR 88 BP 105/58 Temp 37.2°C HGT 10.2

TEWS (circle one): 0 1 2 3 **4** 5 6 7 8 9 10 11 12 13 14 15

Discriminator present? **Yes** No

If yes, write the discriminator: High energy transfer

Final SATS (circle one): Green Yellow **Orange** Red Blue

---

3. You are called to the private residence of a 78 year old female for vomiting and severe abdominal pain (9/10). She looks unwell, and is a known diabetic on insulin. She has not eaten properly for 2 days. You bring her into the ED in a wheelchair.
Vital signs: RR 28 HR 95 BP 147/75 Temp 36°C HGT 9.3

**TEWS (circle one):**	0 1 2 **3** 4 5 6 7 8 9 10 11 12 13 14 15
**Discriminator present?**	**Yes** No
**If yes, write the discriminator:**	Severe Pain
**Final SATS (circle one):**	Green Yellow **Orange** Red Blue

---

4. You are called to the private residence of a 56 year old female. Her husband answers the door. She appears exhausted, groaning, weak and unable to walk. Her face is pale and sweaty. She says she is short of breath and in severe body pain.
Vital signs: RR 29 HR 123 BP 84/57 Temp 37.5°C HGT 8.3 O2 Sat 96%

**TEWS (circle one):**	0 1 2 3 4 5 6 **7** 8 9 10 11 12 13 14 15
**Discriminator present?**	**Yes** No
**If yes, write the discriminator:**	Shortness of breath - acute
**Final SATS (circle one):**	Green Yellow Orange **Red** Blue

---

5. You are called to the private residence of a 62 year old female with an arm injury. She states that she fell over her cat this morning and is now in moderate pain (6/10). She is mobile, and it appears that she has fractured her left wrist.
Vital signs: RR 20 HR 52 BP 121/78 Temp 36.9°C

**TEWS (circle one):**	0 1 **2** 3 4 5 6 7 8 9 10 11 12 13 14 15
**Discriminator present?**	**Yes** No
**If yes, write the discriminator:**	Moderate Pain, Fracture - Closed
**Final SATS (circle one):**	Green **Yellow** Orange Red Blue

---

6. You are called to the workplace of a 34 year old male. He is complaining of severe abdominal pain and walks to the ambulance.
Vital signs: RR 12 HR 88 BP 157/97 Temp 37.1°C

**TEWS (circle one):**	**0** 1 2 3 4 5 6 7 8 9 10 11 12 13 14 15
**Discriminator present?**	**Yes** No
**If yes, write the discriminator:**	Severe pain
**Final SATS (circle one):**	Green Yellow **Orange** Red Blue

---

7. You are called to the private residence of a 19 year old female. She tells you "My boyfriend had too much alcohol last night and hit me over the head". She is mobile, and there is some dry blood around the laceration on her head, but it appears to be superficial.
Vital signs: RR 12 HR 88 BP 106/68 Temp 36.4°C HGT 5.3

**TEWS (circle one):**	0 **1** 2 3 4 5 6 7 8 9 10 11 12 13 14 15
**Discriminator present?**	Yes **No**
**If yes, write the discriminator:**	
**Final SATS (circle one):**	**Green** Yellow Orange Red Blue

8. You are called to the work place of a 27 year old male. He reports that his foot got caught in a machine and complains of moderate pain. His foot is covered in blood, and you help him into a wheelchair.
Vital signs: RR 18 HR 49 BP 135/69 Temp 36°C

**TEWS (circle one):**	0 1 2 3 **4** 5 6 7 8 9 10 11 12 13 14 15
**Discriminator present?**	**Yes** No
**If yes, write the discriminator:**	Moderate pain
**Final SATS (circle one):**	Green **Yellow** Orange Red Blue

9. You are called to the workplace of a 23 year old healthy looking male. He tells you "I was grinding an axe 2 days ago, and I think a piece of metal landed in my left eye". He walks to the ambulance and claims that this foreign body in his eye is causing moderate pain.
Vital signs: RR 12 HR 59 BP 138/88 Temp 36.3°C

**TEWS (circle one):**	0 **1** 2 3 4 5 6 7 8 9 10 11 12 13 14 15
**Discriminator present?**	**Yes** No
**If yes, write the discriminator:**	Moderate pain
**Final SATS (circle one):**	Green **Yellow** Orange Red Blue

10. You are called to the day hospital for a 43 year old male who states he was assaulted 3 days ago and has moderate pain above his left eye. He has been referred for a review and has not experienced any new trauma since he was hit with a stick on his eyebrow.
Vital signs: RR 20 HR 55 BP 146/43 Temp 36°C

**TEWS (circle one):**	0 **1** 2 3 4 5 6 7 8 9 10 11 12 13 14 15
**Discriminator present?**	**Yes** No
**If yes, write the discriminator:**	Moderate pain
**Final SATS (circle one):**	Green **Yellow** Orange Red Blue

11. You are called to the private residence of a 93 year old female. She is very well looking and states, "I was shifting my bed and now have moderate left sided chest pain." She lives alone and has no other medical problems. You assist her with a wheelchair.
Vital signs: RR 12 HR 83 BP 171/89 Temp 36.8°C

**TEWS (circle one):**	0 1 **<u>2</u>** 3 4 5 6 7 8 9 10 11 12 13 14 15
**Discriminator present?**	**<u>Yes</u>** No
**If yes, write the discriminator:**	Chest pain
**Final SATS (circle one):**	Green Yellow **<u>Orange</u>** Red Blue

---

12. You are called to the scene for a 32 year old male. He is very vocal and stating that he was allegedly assaulted. He complains of severe chest pain after being kicked in the left rib and has a productive cough. He walks to the ambulance.
Vital signs: RR 20 HR 106 BP 173/101 Temp 35°C

**TEWS (circle one):**	0 1 2 **<u>3</u>** 4 5 6 7 8 9 10 11 12 13 14 15
**Discriminator present?**	**<u>Yes</u>** No
**If yes, write the discriminator:**	Severe pain, chest pain
**Final SATS (circle one):**	Green Yellow **<u>Orange</u>** Red Blue

---

13. You are called to the private residence of a 49 year old female. She has been very depressed and suicidal and overdosed on medication. She is alert and mobile but very despondent.
Vital signs: RR 22 HR 90 BP 135/64 Temp 36.9°C HGT 6.1

**TEWS (circle one):**	0 1 **<u>2</u>** 3 4 5 6 7 8 9 10 11 12 13 14 15
**Discriminator present?**	**<u>Yes</u>** No
**If yes, write the discriminator:**	Poisoning/Overdose
**Final SATS (circle one):**	Green Yellow **<u>Orange</u>** Red Blue

---

14. You are called to the private residence of a 44 year old female who walks to the door. She is complaining of moderate anal pain due to thrombosed external piles.
Vital signs: RR 20 HR 70 BP 154/94 Temp 36.9°C

**TEWS (circle one):**	0 **<u>1</u>** 2 3 4 5 6 7 8 9 10 11 12 13 14 15
**Discriminator present?**	**<u>Yes</u>** No
**If yes, write the discriminator:**	Moderate pain
**Final SATS (circle one):**	Green **<u>Yellow</u>** Orange Red Blue

---

15. You are called to the private residence of a 35 year old male. He is immobile and has a past history of pulmonary TB. He is a known ethanol abuser and has been confused and unable to speak for the past 2 days. He responds to pain only.
Vital signs: RR 23 HR 148 BP 129/83 Temp 36.8°C

TEWS (circle one): 0 1 2 3 4 5 6 7 8 **9** 10 11 12 13 14 15
Discriminator present? **Yes** No
If yes, write the discriminator: Level of consciousness reduced/confused
Final SATS (circle one): Green Yellow Orange **Red** Blue

---

16. You are called to the private residence of a 37 year old male. He is complaining of moderate back pain. He is not able to walk and is feeling extremely weak. He is otherwise well.
Vital signs: RR 17 HR 76 BP 121/75 Temp 36°C HGT 4.2

TEWS (circle one): 0 1 2 **3** 4 5 6 7 8 9 10 11 12 13 14 15
Discriminator present? **Yes** No
If yes, write the discriminator: Moderate pain
Final SATS (circle one): Green **Yellow** Orange Red Blue

---

17. You are called to a private residence by police for an aggressive 23 year old female. She is mobile, alert but crying and very disruptive and psychotic.
Vital signs: RR 12 HR 91 BP 151/89 Temp 36.6°C O2 Sat 100%

TEWS (circle one): **0** 1 2 3 4 5 6 7 8 9 10 11 12 13 14 15
Discriminator present? **Yes** No
If yes, write the discriminator: Aggression
Final SATS (circle one): Green Yellow **Orange** Red Blue

---

18. You are called to the private residence of a frail and dehydrated 79 year old female. She complains of persistent vomiting and abdominal pain. She is a known diabetic and has a past history of renal failure. You bring her into the ED in a wheelchair.
Vital signs: RR 18 HR 76 BP 209/76 Temp 37.4°C HGT 7.8

TEWS (circle one): 0 1 2 3 **4** 5 6 7 8 9 10 11 12 13 14 15
Discriminator present? **Yes** No
If yes, write the discriminator: Abdominal pain, Persistent vomiting
Final SATS (circle one): Green **Yellow** Orange Red Blue

---

19. You are called to the jail to assess a 29 year old male prisoner who complains of shortness of breath and weight loss. He appears pale, tired, and weak and has a history of Pulmonary TB.
Vital signs: RR 37 HR 135 BP 103/67 Temp 36.7°C HGT 5.4

**TEWS (circle one):**	0 1 2 3 4 5 **6** 7 8 9 10 11 12 13 14 15
**Discriminator present?**	**Yes** No
**If yes, write the discriminator:**	Shortness of breath - acute
**Final SATS (circle one):**	Green Yellow **Orange** Red Blue

---

20. You arrive at an industrial building, and your patient walks to the ambulance. He is a 40 year old male with a history of asthma, and he has been working with acid earlier in the day. He appears tachypneic (breathing fast), and the symptoms have been worsening over the last several hours.
Vital signs: RR 19 HR 93 BP 133/72 Temp 37.2°C HGT 4.9

**TEWS (circle one):**	0 **1** 2 3 4 5 6 7 8 9 10 11 12 13 14 15
**Discriminator present?**	**Yes** No
**If yes, write the discriminator:**	Shortness of breath – acute, Poisoning/overdose
**Final SATS (circle one):**	Green Yellow **Orange** Red Blue

---

21. You arrive at a private residence and are led by family to a 70 year old male laying in bed. He responds to voice and appears to be short of breath. He is normally independent, and this is the first time family has seen him in 2 days.
Vital signs: RR 44 HR 163 BP 113/69 Temp 36.4°C HGT 12.4

**TEWS (circle one):**	0 1 2 3 4 5 6 7 8 **9** 10 11 12 13 14 15
**Discriminator present?**	**Yes** No
**If yes, write the discriminator:**	Level of consciousness reduced
**Final SATS (circle one):**	Green Yellow Orange **Red** Blue

---

22. You are called to the shopping mall for an 18 year old female accompanied by her mother. The patient fainted and fell to the ground. She is now bleeding from her right eardrum and vomiting persistently. She is alert but looks extremely pale and tells you she "feels awful" and vomits. She requires assistance to stand up. The patient informs you she is 13 weeks pregnant.
Vital signs: RR 17 HR 80 BP 97/61 Temp 37°C HGT 5.5

**TEWS (circle one):**	0 1 2 3 **4** 5 6 7 8 9 10 11 12 13 14 15
**Discriminator present?**	**Yes** No
**If yes, write the discriminator:**	Persistent vomiting, Pregnancy & trauma
**Final SATS (circle one):**	Green **Yellow** Orange Red Blue

---

23. You are called to a private residence of a 40 year old female who walks out to the ambulance complaining of moderate lower abdominal pain. She has a heavy PV bleed but feels well and appears comfortable.
Vital signs: RR 18 HR 85 BP 148/89 Temp 36.5°C

**TEWS (circle one):**	0 **1** 2 3 4 5 6 7 8 9 10 11 12 13 14 15
**Discriminator present?**	**Yes** No
**If yes, write the discriminator:**	Moderate pain, abdominal pain
**Final SATS (circle one):**	Green **Yellow** Orange Red Blue

---

24. You are called to a private clinic for a 76 year old female complaining of chest pain. She is mobile and in no distress and says that she has experienced sharp central pain since last night.
Vital signs: RR 16 HR 55 BP 150/68 Temp 36.5°C HGT 7.2

**TEWS (circle one):**	0 **1** 2 3 4 5 6 7 8 9 10 11 12 13 14 15
**Discriminator present?**	**Yes** No
**If yes, write the discriminator:**	Chest pain
**Final SATS (circle one):**	Green Yellow **Orange** Red Blue

---

25. You are called to the private residence of 58 year old male who has been fitting for 6 hours on and off pre-arrival. He is alert but really doesn't feel so well. You bring him into the ED on a stretcher.
Vital signs: RR 23 HR 67 BP 126/81 Temp 36.9°C HGT 5.2 O2 Sat 100%

**TEWS (circle one):**	0 1 2 3 **4** 5 6 7 8 9 10 11 12 13 14 15
**Discriminator present?**	**Yes** No
**If yes, write the discriminator:**	Seizure – post ictal
**Final SATS (circle one):**	Green Yellow **Orange** Red Blue

---

26. You are called to the scene and find a 42 year old homeless male who is a known non compliant epileptic. He claims to have fallen on his head and there is dry blood around the 2cm laceration on his head. He is unkempt and smells of alcohol. You must assist him with a wheelchair.
Vital signs: RR 24 HR 78 BP 111/69 Temp 36°C HGT 11.8

**TEWS (circle one):**	0 1 2 3 **4** 5 6 7 8 9 10 11 12 13 14 15
**Discriminator present?**	Yes **No**
**If yes, write the discriminator:**	
**Final SATS (circle one):**	Green **Yellow** Orange Red Blue

---

27. You are called to the private residence of a 20 year old pregnant female. She has a history of abdominal pain during pregnancy but is otherwise healthy. She walks out the ambulance and states she would like a check up.
Vital signs: RR 12 HR 87 BP 126/68 Temp 36.8°C

**TEWS (circle one):**	**<u>0</u>** 1 2 3 4 5 6 7 8 9 10 11 12 13 14 15
**Discriminator present?**	Yes **<u>No</u>**
**If yes, write the discriminator:**	
**Final SATS (circle one):**	**<u>Green</u>** Yellow Orange Red Blue

---

28. You are called to the private residence of a talkative 57 year old male who walks to the door with extremely swollen legs and a distended abdomen. He has a history of hypertension and COAD but nothing else of note. He complains of moderate pain (5/10) and was well until his legs swelled up.
Vital signs: RR 16 HR 106 BP 126/77 Temp 36.8°C HGT 11.2

**TEWS (circle one):**	0 1 **<u>2</u>** 3 4 5 6 7 8 9 10 11 12 13 14 15
**Discriminator present?**	**<u>Yes</u>** No
**If yes, write the discriminator:**	Moderate pain
**Final SATS (circle one):**	Green **<u>Yellow</u>** Orange Red Blue

---

29. You are called to a private clinic for a 24 year old female. She is sweaty and appears to be in a lot of discomfort. She states that she is feeling really unwell, has severe backache and fever. She feels hot to touch. She has felt really unwell for 2 days now and walks out to the ambulance.
Vital signs: RR 16 HR 114 BP 95/60 Temp 40°C

**TEWS (circle one):**	0 1 2 3 4 5 **<u>6</u>** 7 8 9 10 11 12 13 14 15
**Discriminator present?**	**<u>Yes</u>** No
**If yes, write the discriminator:**	Severe pain
**Final SATS (circle one):**	Green Yellow **<u>Orange</u>** Red Blue

---

30. You are called to the work place of a 32 year old male with a swollen left eye and face. He complains of moderate pain and explains that he has a 2 day old eye injury from a blunt assault, which was treated in the hospital. He states his vision is getting worse and walks with you to the ambulance.
Vital signs: RR 12 HR 67 BP 122/73 Temp 36.5°C

**TEWS (circle one):**	**<u>0</u>** 1 2 3 4 5 6 7 8 9 10 11 12 13 14 15
**Discriminator present?**	**<u>Yes</u>** No
**If yes, write the discriminator:**	Moderate pain
**Final SATS (circle one):**	Green **<u>Yellow</u>** Orange Red Blue

---

31. You are called to the private residence of a 40 year old woman. She walks to open the door and appears weak. She states that she has been vomiting fresh blood.
Vital signs: RR 42 HR 131 BP 127/74 Temp 38°C HGT 7

**TEWS (circle one):**	0 1 2 3 4 5 **6** 7 8 9 10 11 12 13 14 15
**Discriminator present?**	**Yes** No
**If yes, write the discriminator:**	Vomiting – fresh blood
**Final SATS (circle one):**	Green Yellow **Orange** Red Blue

32. You are called to the private residence of a 31 year old female. She tells you "Someone pushed me into a fire 1 hour ago" and complains of a burn surrounding her entire right lower leg. You assist her into the ambulance.
Vital signs: RR 16 HR 80 BP 125/65 Temp 36.8°C

**TEWS (circle one):**	0 1 2 **3** 4 5 6 7 8 9 10 11 12 13 14 15
**Discriminator present?**	**Yes** No
**If yes, write the discriminator:**	Burn - circumferential
**Final SATS (circle one):**	Green Yellow **Orange** Red Blue

33. You are called to the scene for a 32 year old male who was stabbed. He is bleeding from his left parietal lacerations but able to stop the bleeding with direct pressure. He is walking around the scene and states he had an argument with someone who then tried to stab him.
Vital signs: RR 18 HR 61 BP 126/81 Temp 36°C

**TEWS (circle one):**	0 1 **2** 3 4 5 6 7 8 9 10 11 12 13 14 15
**Discriminator present?**	**Yes** No
**If yes, write the discriminator:**	Haemorrhage - controlled
**Final SATS (circle one):**	Green **Yellow** Orange Red Blue

34. You are called to the scene of an accident for a 46 year old male in a pedestrian vehicle accident 1 hour ago. He was struck by a truck going over 100-Km/Hr. He appears to have multiple devastating injuries to his head, chest, arms, and legs. He is pulseless and not breathing.
Vital signs: RR 0 HR 0 BP 0

**TEWS (circle one):**	0 1 2 3 4 5 6 7 8 9 10 11 12 **13** 14 15
**Discriminator present?**	Yes **No**
**If yes, write the discriminator:**	
**Final SATS (circle one):**	Green Yellow Orange Red **Blue**

35. You are called to the workplace of a 39 year old male. He experienced blunt trauma to his right hand, looks healthy and is able to walk.
Vital signs: RR 20 HR 45 BP 130/71 Temp 36°C

**TEWS (circle one):**	0 1 2 **3** 4 5 6 7 8 9 10 11 12 13 14 15
**Discriminator present?**	Yes **No**
**If yes, write the discriminator:**	
**Final SATS (circle one):**	Green **Yellow** Orange Red Blue

---

36. You are called to the private residence of a 27 year old female. She states that she has had heavy PV bleeding for past days with clots. Her pregnancy test came out negative and she appears pale but states she is in no pain. She had a miscarriage about 2 months ago.
Vital signs: RR 20 HR 66 BP 114/74 Temp 36.5°C HGT 4.9

**TEWS (circle one):**	0 **1** 2 3 4 5 6 7 8 9 10 11 12 13 14 15
**Discriminator present?**	Yes **No**
**If yes, write the discriminator:**	
**Final SATS (circle one):**	**Green** Yellow Orange Red Blue

---

37. You are called to the scene for a 46 year old female who fell. She is mobile and complaining of severe pain in her arm. She has no history of other medical problems.
Vital signs: RR 17 HR 82 BP 164/110 Temp 36.4°C HGT 5

**TEWS (circle one):**	0 1 **2** 3 4 5 6 7 8 9 10 11 12 13 14 15
**Discriminator present?**	**Yes** No
**If yes, write the discriminator:**	Severe pain
**Final SATS (circle one):**	Green Yellow **Orange** Red Blue

---

38. You are called to the work place of a fit and healthy looking 35 year old male. He stated he hit his thumb with a hammer. He does not appear to have a dislocation, is mobile and does not appear distressed.
Vital signs: RR 20 HR 68 BP 145/92 Temp 36°C HGT 4.9

**TEWS (circle one):**	0 1 **2** 3 4 5 6 7 8 9 10 11 12 13 14 15
**Discriminator present?**	Yes **No**
**If yes, write the discriminator:**	
**Final SATS (circle one):**	**Green** Yellow Orange Red Blue

---

39. You are called to the private residence of a 29 year old male complaining of severe chest pain. He is mobile but looks tired.
Vital signs: RR 16 HR 58 BP 112/61 Temp 36.2°C

**TEWS (circle one):**	0 **1** 2 3 4 5 6 7 8 9 10 11 12 13 14 15
**Discriminator present?**	**Yes** No
**If yes, write the discriminator:**	Severe pain, chest pain
**Final SATS (circle one):**	Green Yellow **Orange** Red Blue

---

40. You are called to the private residence of a 17 year old female who complains of hoarseness. She is mobile and does not appear to be distressed.
Vital signs: RR 16 HR 62 BP 121/71 Temp 36.5°C

**TEWS (circle one):**	0 **1** 2 3 4 5 6 7 8 9 10 11 12 13 14 15
**Discriminator present?**	Yes **N**o
**If yes, write the discriminator:**	
**Final SATS (circle one):**	**Green** Yellow Orange Red Blue

---

41. You are called to the scene of a 33 year old male. He was stabbed over his left scapula and in his right leg. He is cold and sweaty and responds to voice only sitting in a wheelchair. He appears to be in severe pain.
Vital signs: RR 30 HR 100 BP 116/74 Temp 37°C

**TEWS (circle one):**	0 1 2 3 4 5 6 **7** 8 9 10 11 12 13 14 15
**Discriminator present?**	**Yes** No
**If yes, write the discriminator:**	Level of consciousness - reduced
**Final SATS (circle one):**	Green Yellow Orange **Red** Blue

---

42. You are called to the private residence of a 39 year old female. She stated that she is experiencing the worst abdominal pain ever. She is mobile and alert, but appears to be in extreme agony. She has had a previous evacuation of an ulcer.
Vital signs: RR 12 HR 91 BP 134/91 Temp 37°C HGT 4.5

**TEWS (circle one):**	**0** 1 2 3 4 5 6 7 8 9 10 11 12 13 14 15
**Discriminator present?**	**Yes** No
**If yes, write the discriminator:**	Severe pain
**Final SATS (circle one):**	Green Yellow **Orange** Red Blue

---

43. You are called to the private residence of a 48 year old female who complains of headache and weakness in her left arm. She is mobile and has had a previous CVA.
Vital signs: RR 20 HR 117 BP 205/136 Temp 36°C HGT 7.9

TEWS (circle one): 0 1 2 3 4 **5** 6 7 8 9 10 11 12 13 14 15
Discriminator present? **Yes** No
If yes, write the discriminator: Focal neurology - acute
Final SATS (circle one): Green Yellow **Orange** Red Blue

---

44. You are called to the private residence of a 30 year old female. She appears fragile laying in bed. She presents with right sided weakness and aphagia. She is HIV+ and reacts to pain only.
Vital signs: RR 24 HR 98 BP 106/78 Temp 36.9°C HGT 5.2

TEWS (circle one): 0 1 2 3 4 5 **6** 7 8 9 10 11 12 13 14 15
Discriminator present? **Yes** No
If yes, write the discriminator: Focal neurology – acute, Level of consciousness reduced
Final SATS (circle one): Green Yellow **Orange** Red Blue

---

45. You are called to the scene for a 65 year old male with a gun shot wound to the abdomen. You place him on a stretcher. He complains of mild pain over the right iliac fossa. There is blood on his abdomen over the entry wound.
Vital signs: RR 20 HR 84 BP 147/91 Temp 37°C HGT 5.5

TEWS (circle one): 0 1 2 3 **4** 5 6 7 8 9 10 11 12 13 14 15
Discriminator present? **Yes** No
If yes, write the discriminator: High energy transfer
Final SATS (circle one): Green Yellow **Orange** Red Blue

---

46. You are called to a private residence of a 40 year old woman. You find the patient drowsy and she states she overdosed on cocaine and benzodiazepines. She is a known drug addict with a history of depression. She is alert but uncooperative and a little confused.
Vital signs: RR 12 HR 65 BP 120/80 Temp 37°C

TEWS (circle one): 0 1 **2** 3 4 5 6 7 8 9 10 11 12 13 14 15
Discriminator present? **Yes** No
If yes, write the discriminator: Poisoning/overdose
Final SATS (circle one): Green Yellow **Orange** Red Blue

---

47. You are called to a private residence and find a 42 year old male complaining of "The worst pain I have ever felt" and rates it as 9 on a scale of 1 to 10. The pain is in his left buttock and he has a history of buttock abscesses and pulmonary TB.
Vital signs: RR 16 HR 73 BP 150/85 Temp 37°C HGT 5.8

**TEWS (circle one):**	0 **1** 2 3 4 5 6 7 8 9 10 11 12 13 14 15
**Discriminator present?**	**Yes** No
**If yes, write the discriminator:**	Severe pain
**Final SATS (circle one):**	Green Yellow **Orange** Red Blue

---

48. You are called to a street corner for a 34 year old male who reports "They just started assaulting me - hitting and kicking my chest". You found him walking and complaining of severe chest pain with no other obvious injuries.
Vital signs: RR 24 HR 108 BP 116/82 Temp 36.5°C

**TEWS (circle one):**	0 1 2 3 **4** 5 6 7 8 9 10 11 12 13 14 15
**Discriminator present?**	**Yes** No
**If yes, write the discriminator:**	Severe pain, chest pain
**Final SATS (circle one):**	Green Yellow **Orange** Red Blue

---

49. You arrive at a private residence and family leads you to an unresponsive 33 year old male. Family states they found him unconscious in bed this morning. He appears to going into episodic convulsions. He smells of alcohol, is unkempt and has been incontinent of urine.
Vital signs: RR 26 HR 138 BP 79/60 Temp 37°C HGT 3.1 O2 Sats 95%

**TEWS (circle one):**	0 1 2 3 4 5 6 7 8 9 10 11 **12** 13 14 15
**Discriminator present?**	**Yes** No
**If yes, write the discriminator:**	Seizure - current
**Final SATS (circle one):**	Green Yellow Orange **Red** Blue

---

50. You are called to an old age home to transport a 91 year old female. She walks with assistance and complains of moderate left hip and left ankle pain. She has not experienced any recent trauma but fell 5 years ago and has an old hip injury. She is very chatty but a little confused, which is normal for her apparently.
Vital signs: RR 16 HR 65 BP 127/64 Temp 37.4°C HGT 4.1

**TEWS (circle one):**	0 1 **2** 3 4 5 6 7 8 9 10 11 12 13 14 15
**Discriminator present?**	**Yes** No
**If yes, write the discriminator:**	Moderate pain
**Final SATS (circle one):**	Green **Yellow** Orange Red Blue

END VIGNETTES.

Thank you for participating! Please leave us any comments here:

____

____

____

____

____

____
